# Cyanidin-3-O-glucoside plays a protective role against renal ischemia/ reperfusion injury via the JAK/STAT pathway

**DOI:** 10.1590/acb381023

**Published:** 2023-05-01

**Authors:** Yufeng Xiong, Jun Jian, Honglin Yu, Jiejun Wu, Hu Mao, Ruikang Feng, Lei Wang, Yonghong Jian, Xiuheng Liu

**Affiliations:** 1 Renmin Hospital of Wuhan University – Department of Urology – Wuhan (Hubei), China.; 2 Renmin Hospital of Wuhan University – Institute of Urologic Disease – Wuhan (Hubei), China.; 3 University of Science and Technology of China, The First Affiliated Hospital – Department of Radiology – Hefei (Anhui), China.; 4 Renmin Hospital of Wuhan University – Department of Nephrology – Wuhan (Hubei), China.

**Keywords:** Oxidative Stress, Apoptosis, Ischemia, Reperfusion, Apoptosis, Endoplasmic Reticulum Stress

## Abstract

**Purpose:**

To investigate the role of cyanidin-3-O-glucoside (C3G) in renal ischemia/reperfusion (I/R) injury and the potential mechanisms.

**Methods:**

Mouse models were established by clamping the left renal vessels, and in vitro cellular models were established by hypoxic reoxygenation.

**Results:**

Renal dysfunction and tissue structural damage were significantly higher in the I/R group. After treatment with different concentrations of C3G, the levels of renal dysfunction and tissue structural damage decreased at different levels. And its protective effect was most pronounced at 200 mg/kg. The use of C3G reduced apoptosis as well as the expression of endoplasmic reticulum stress (ERS)-related proteins. Hypoxia/reoxygenation (H/R)-induced apoptosis and ERS are dependent on oxidative stress in vitro. In addition, both AG490 and C3G inhibited the activation of JAK/STAT pathway and attenuated oxidative stress, ischemia-induced apoptosis and ERS.

**Conclusions:**

The results demonstrated that C3G blocked renal apoptosis and ERS protein expression by preventing reactive oxygen species (ROS) production after I/R via the JAK/STAT pathway, suggesting that C3G may be a potential therapeutic agent for renal I/R injury.

## Introduction

Renal ischemia/reperfusion (I/R) injury is a common clinical pathology in which renal dysfunction and tissue damage are further worsened after restoration of renal tissue perfusion on the basis of reduced or blocked blood perfusion. This injury is very common in surgery and may be caused by partial nephrectomy, renal transplantation and partial vascular surgery. I/R injury is also associated with several high-risk factors and is a common pathophysiological process that results in impairment of vital organ function from many different causes^1^^-^ 4. Renal I/R leads to apoptosis and tissue necrosis, resulting in acute tubular necrosis, which impairs renal function and results in a high mortality rate of the disease^5^. Although significant progress has been made in recent years in the treatment and prevention of acute kidney injury (AKI), there is still a lack of effective drug therapy^6^. A recent study shows that abnormal apoptosis of renal tubular epithelial cells, oxidative stress, and endoplasmic reticulum stress (ERS) may influence the occurrence and progression of AKI^7^.With the increasing importance of preventing AKI, it is essential to develop new therapeutic agents and interventions to prevent renal damage caused by renal I/R.

Anthocyanins are flavonoid phytochemicals widely distributed in plants. Anthocyanins extracted from food or plants often contain different anthocyanin elements, such as cyanidin, pelargonidin, petunidin, and malvidin^8^.These monomeric anthocyanins with different structures have different physiological activities. In recent years, the anthocyanins from various plants, especially black beans, have been extensively studied and their excellent antioxidant, enzyme inhibitory, vasoprotective, anti-inflammatory, and antitumor activities have been confirmed^9^. Among them, cyanidin-3-O-glucoside (C3G) is the main active ingredient. Besides, C3G is extracted from natural plants, and black bean, which is the main source, is a traditional crop grown in China and has a long history of edible and medicinal use.

Signal transducer and activator of transcription protein (STAT) is phosphorylated and dimerized by Janus kinase (JAK), and then translocated through the nuclear membrane into the nucleus to regulate the expression of related genes. This pathway is known as the JAK/STAT signaling pathway. This signaling pathway is associated with various bodily functions and is involved in several important biological processes including cell proliferation, differentiation, apoptosis, inflammatory response, immune regulation, and hematopoiesis^10^^,^ 11. JAK/STAT, as one of the most important signaling pathways, directly regulates the communication between transmembrane receptors and the nucleus. First, cytokines bind and induce dimerization of the corresponding receptors, which allows JAK kinase to couple with phosphorylated receptors. Second, tyrosine residues on the catalytic domain of the receptor are phosphorylated and form docking sites for surrounding amino acids, so that STAT proteins with SH2 structural domains are recruited to the docking sites. Third, STAT is phosphorylated and activated to form a dimer. Finally, dimerized STATs in the cytoplasm are transferred to the nucleus by binding to specific DNA elements to regulate the expression of cytokine-responsive genes^10^^,^ 12 ^,^ 13.

Recent evidence suggests that apoptosis of cardiomyocytes after myocardial I/R injury is inhibited by inhibiting the JAK/STAT signaling pathway. The JAK/STAT pathway may be considered as a potential target for ameliorating oxidative stress processes in focal cerebral ischemic injury^14^^,^ 15. However, up to now, the role of cyanidin-3-O-glucoside, which has strong antioxidant activity, in renal I/R injury has not been thoroughly investigated, and whether the JAK/STAT signaling pathway is also involved in the process of renal ischemic injury and whether it is a potential target for the action of C3G also deserves in-depth study and exploration.

## Methods

### Experimental animals, renal I/R model and dosage information

Adult male C57Bl/6 mice (20–25 g) were provided by the Experimental Animal Center of the Medical College of Wuhan University. The project was approved by the Wuhan University Laboratory Animal Committee. Mice were anesthetized using inhaled isoflurane (5%, 1 L/min), then placed on a thermostatic table to maintain body temperature at 37 °C. A right nephrectomy was performed after entering the abdominal cavity through a median abdominal incision, and then the left kidney was blocked from perfusion by closing the blood vessels with a nontraumatic vascular clamp; successful blockage was demonstrated by observing the kidney turning dark red. After 30 min, the nontraumatic vascular clamp was released and the kidney was observed to turn bright red, which proved successful reperfusion.

All mice were randomly divided into different treatment groups (Sham, I/R, I/R+DMSO, I/R+50 mg/kg, I/R+100 mg/kg, I/R+200 mg/kg, 5 mice per group). In the Sham group, only the right nephrectomy was performed. In the I/R+C3G group, the surgical operation was the same as in the I/R group, but different doses of C3G (50, 100, and 200 mg/kg) were administered by gavage daily for 2 weeks before model establishment. Mice in the Sham and I/R groups received equal amounts of saline gavage. The treatment in the I/R+DMSO group was the same as in the I/R+C3G group, and the gavage was changed to equal amounts of 1% DMSO.

To consume equivalent amounts of anthocyanins, adults need to consume about 60–100 g of dried bilberry or 1 L of bilberry juice per day equivalent to the 50 mg/kg dose used in mouse models^16^. In several clinical trials, a dose of 200 mg/kg corresponds to daily anthocyanin supplementation^17^.

### Cell culture and the cell hypoxia/reoxygenation (H/R) model

Human renal proximal tubular epithelial cell line (HK-2) was obtained from the American Type Culture Collection (ATCC, USA); HK-2 cells were cultured in DMEM medium containing 0.05 mg/mL bovine pituitary extract, 50 ng/mL human recombinant epidermal growth factor, 100 U/mL penicillin, 100 μg/mL streptomycin, and 10% fetal bovine serum at 37 °C in a 5% CO_2_ and 95% air environment. The cell H/R model was established as following methods. First, HK-2 cells were incubated under hypoxic conditions (1% O_2_, 94% N_2_ and 5% CO_2_) in nutrient-free (glucose-free, serum-free) medium for 12 h to induce hypoxic damage. Then, the medium was changed again and the plates were moved to a normoxic cell culture incubator (5% CO_2_ and 95% air) for 6 h. Control cells were cultured in complete medium in a conventional incubator (5% CO_2_ and 95% air).

### Western blot analysis

HK-2 cells and kidney tissues were homogenized in radioimmunoprecipitation assay buffer (Beyotime, Nanjing, China) containing protease inhibitors to lyse the cells to obtain total proteins. After collecting the proteins in each sample separately, the proteins were separated by 10% sodium dodecyl sulfate-polyacrylamide gel electrophoresis (SDS-PAGE) and then transferred to PVDF membranes in an ice-water bath and incubated with primary antibody at 4 °C for 12 h after being closed with 5% skim milk for 1 h at room temperature. Primary antibody was used at the following dilutions: Bax (1:200, Cell Signaling, 2772); Bcl-2 (1:1000, Abcam, Ab196495); CHOP (1:1000, Cell Signaling, 2895); GRP78 (1: 1000, Abcam, Ab21685) and GAPDH (1:1000, Hangzhou Jiahe Biotechnology Co., AB-PR 001);JAK2(1:5000, Abcam, Ab108596);p-JAK2(1:1000, Abcam, Ab32101);STAT3(1:1000, Abcam, Ab68153);p-STAT3(1:1000, Abcam, Ab267373). The membranes were then washed three times with TBST for 20 min each, incubated for 2 h at room temperature with appropriate secondary antibodies, then washed three times with TBST for 10 min each, and finally the western blots were visualized using chemiluminescent HRP substrate (Millipore, Billerica, MA, USA). Data analysis was performed using Image J software (NIH, USA) to quantify protein levels.

### Histological staining

After fixing and embedding the kidney tissue and cutting into 4 μm thick sections, they were stained with hematoxylin and eosin (H&E). The specimens were evaluated by two experienced renal pathologists who unknowingly rated the extent of kidney damage according to the rating criteria of Jablonski et al.^18^: Obvious tubular dilatation or cell flattening (1 pt); tubular pattern in the tubule (2 pts); detached or necrotic cells in the tubular lumen, but no tubular pattern or cellular debris (1 pt); granular degeneration of epithelial cells (1 pt); vacuolar degeneration (1 pt); nuclear fixation (1 pt).

### Serum assays

One milliliter of venous blood was taken from mice, and the serum was obtained by centrifugation at room temperature and processed according to the manufacturer’s instructions (Nanjing Jiancheng Co., China). Blood urea nitrogen (BUN) and serum creatinine levels were calculated spectrophotometrically.

### Flow cytometry

Apoptosis was assessed by flow cytometry using the Annexin V-FITC/PI Apoptosis Assay Kit Ltd., # 70-AP101-100 (Multisciences Biotechnology Co., China) according to the manufacturer’s instructions. Briefly, HK-2 cells were washed twice with PBS and stained with a binding buffer containing 5 μL Annexin V-FITC in 10 μL PI for 5 min under light-proof and room temperature conditions. Apoptotic cells were detected by FACS flow cytometry (BD, Germany).

### Malondialdehyde (MDA) and superoxide dismutase (SOD) measurement

MDA concentration (thiobarbituric acid method, Catalog No. A003-1) and SOD activity (xanthine oxidase method, Catalog No. A001-3) were measured according to the kit instructions (Nanjing Jiancheng Company, China).

### Cell viability

Cell viability was assessed using the Cell Counting Kit-8 assay (Beyotime Biotechnology, #C0037) according to the manufacturer’s instructions. Briefly, HK-2 cells were inoculated into 96-well plates, after which they were precultured in an incubator for 24 h (at 37 °C, 5% CO_2_) in order to allow cell apposition. The cells were incubated with different doses of C3G for 24 h after which the H/R model was established. Finally, 10 μL of CCK-8 solution was added to each well and incubated in the incubator for 2 h. The absorbance was measured using a microplate reader (Molecular Devices, USA) at 450 nm.

### Measurement of reactive oxygen species (ROS) production

The Reactive Oxygen Species Assay Kit (Beyotime Biotechnology, #S0063) was used to determine intracellular ROS levels. Briefly, cells from different treatment groups were incubated with 20 µmol/L dichlorodihydrofluorescein diacetate (DCFH-DA) in buffer for 30 min at 37 °C. ROS levels were observed using fluorescence microscopy, and the green fluorescence intensity represents the level of ROS.

### Statistical analysis

The primary outcome was the degree of renal injury after I/R, with Creatinine (Cr) as the primary index. According to the literature, the Cr after I/R surgical operation was 150 ± 5 µmol/L, compared with 20 ± 2 µmol/L in the sham group and 100 ± 5 µmol/L in the drug-treated group. Power analysis was performed analysis with the following parameters: α = 0.05 (bilateral), test efficacy Power (1-β) = 0.9, and the sample size was calculated by PASS 27.0, using the Kruskal–Wallis test method, which showed that, according to Cr, each group 3 mice were required. To compensate for potential missed participants, we enrolled 5 mice in each group.

Statistical analysis was performed using Statistical Package for the Social Sciences (SPSS) 27.0 for Windows (SPSS, Inc., USA). All values are expressed as medians (interquartile range, IQR). The Kruskal–Wallis and Dunn tests were introduced to analyze the differences between experimental groups. P < 0.05 indicates that the differences are statistically significant.

## Results

### C3G attenuated renal I/R injury

**Figure 1:**
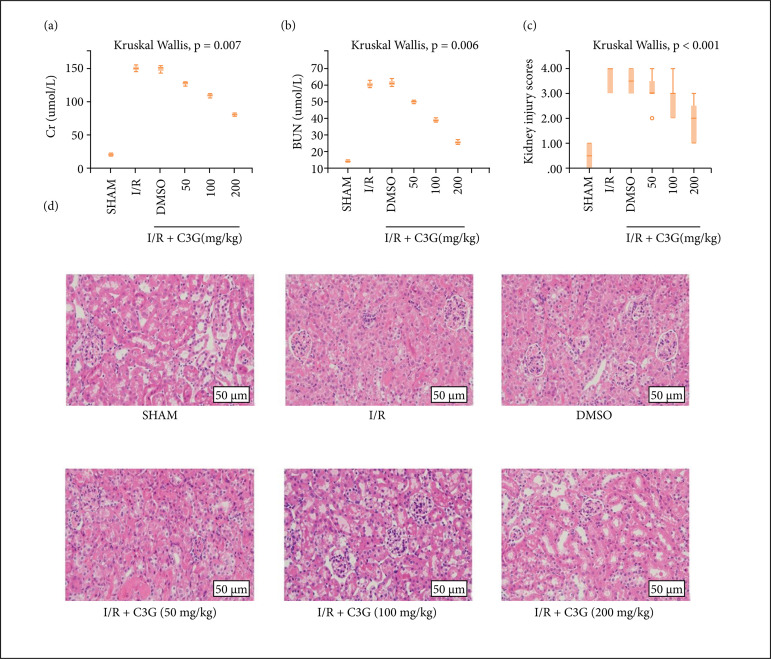
C3G attenuated renal I/R injury. (**a, b**) Protective effects of C3G at doses of 50, 100, and 200 mg/kg on renal function in mice exposed to renal IR (n = 5 each); (**c, d**) Protective effect of C3G at different doses of 50, 100, and 200 mg/kg on renal tissue injury detected by H&E (×400) and randomly selected images from eight independent kidney samples used to quantify renal tubular injury scores. Values are expressed as medians (IQR) on the box plot. P < 0.05 indicates that the differences are statistically significant.

The in vivo renoprotective effect of C3G was first investigated in the mouse I/R model. C57BL/6 mice were randomly assigned to Sham, I/R, I/R + DMSO and I/R + C3G (50, 100 and 200 mg/kg) groups (n = 5 each). Cr and BUN in the blood of mice were first examinated to reflect renal function, and the results showed that mice receiving I/R showed significant renal dysfunction with statistically significant differences (Cr 150.6 ± 3.152 to 21.07 ± 1.220, BUN 59.77± 1.369 to 14.27 ± 0.4978, n = 5, P < 0.05) compared to the Sham group, while mice receiving C3G showed significant improvement in renal function with statistically significant differences (Cr 81.07 ± 1.675 to 150.6 ± 3.152, BUN 25.53 ± 0.9025 to 59.77 ± 1.369, n = 5, P < 0.05) compared to the I/R group. Moreover, the protective effect of C3G was more obvious at a concentration of 200 mg/kg (Fig. 1a, b). The renal tissues of the Sham group behaved normally. However, the kidneys in the I/R group showed acute tubular injury, including brush border loss and tubular dilatation, and C3G protected the tubular epithelium from edema and brush border loss, and this protective effect was highest at a concentration of 200 mg/kg (Fig. 1c, d). These results demonstrated that C3G treatment attenuated renal I/R in a dose-dependent manner. in addition. The dose of 200 mg/kg was selected for all subsequent in vivo experiments.

### C3G reduced apoptosis and ERS in vivo

**Figure 2:**
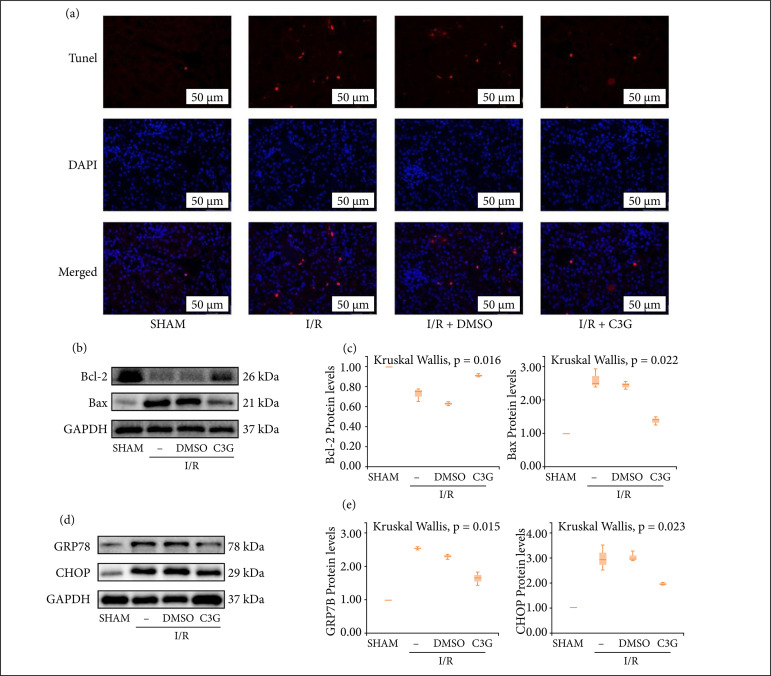
C3G reduced apoptosis and ERS in vivo. **(a)** Representative images of TUNEL staining on kidney sections (×400); **(b)** Protein blot of Bax, Bcl-2 at 24 h of reperfusion; **(c)** and bar graph showed the fold change of Bax, Bcl-2 relative to the SHAM group in three independent samples; **(d)** Protein blots of GRP78, CHOP at 24 h of reperfusion; **(e)** Bar graph showed the change in GRP78, CHOP ploidy. Values are expressed as medians (IQR) on the box plot. P < 0.05 indicates that the differences are statistically significant.

To investigate whether C3G has a protective effect against apoptosis in kidney cells, terminal deoxynucleotidyl transferase dUTP nick end labeling (TUNEL) staining was performed. As shown in Fig. 2a, more TUNEL-positive cells appeared in the renal I/R group compared to the SHAM group, but this was partially eliminated by C3G treatment. In addition, western blotting also showed that C3G treatment reversed the changes in Bax and Bcl-2 expression caused by I/R, and the difference was statistically significant (Bcl-2 0.9133 ± 0.008819 to 0.7267 ± 0.03930, Bax 1.403 ± 0.07265 to 2.650 ± 0.1735, n = 3, P < 0.05) (Fig. 2b, c). Next, it was investigated whether C3G could regulate ERS in I/R mice, and similarly, C3G pretreatment reversed the I/R-induced increase in ERS-related protein GRP78 and CHOP (GRP78 1.650 ± 0.1217 to 2.577 ± 0.03180, CHOP 1.977 ± 0.02728 to 3.010 ± 0.2994, n = 3, P < 0.05) (Fig. 2 d, e). These results suggested that C3G can attenuate I/R-induced apoptosis and ERS.

### H/R-induced apoptosis and ERS depend on oxidative stress in vitro

**Figure 3:**
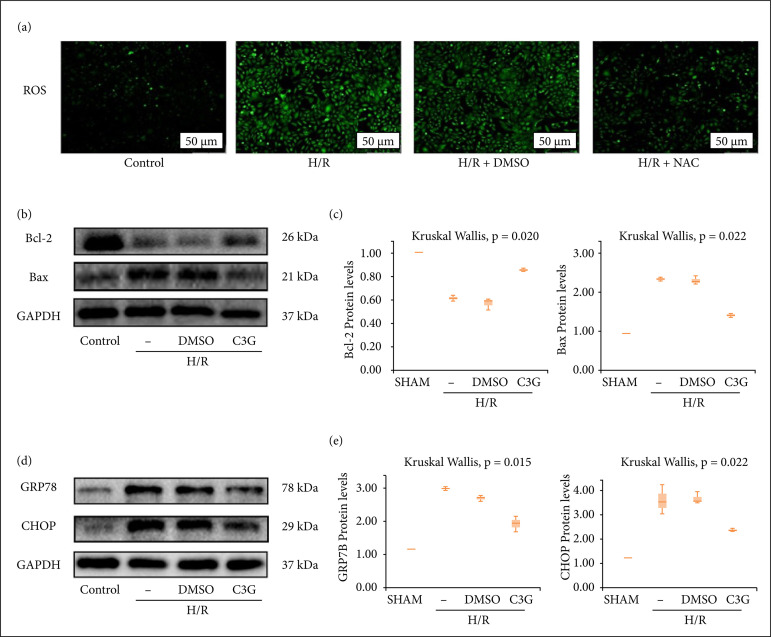
H/R-induced apoptosis and ERS depend on oxidative stress in vitro. HK-2 cells were pretreated with 5 mmol/L NAC for 1 h followed by H/R. **(a)** ROS production observed by fluorescence microscopy (×400); **(b)** Protein blot analysis of Bax, BCL-2 expression; **(c)** Bax, BCL-2 protein expression was quantified by optical densitometry and normalized to the expression of GAPDH from three independent experiments; **(d)** Protein blot analysis of GRP78, CHOP expression; **(e)** GRP78, CHOP protein expression was quantified by optical densitometry normalized to the expression of GAPDH from three independent experiments. Values are expressed as medians (IQR) on the box plot. p < 0.05 indicates that the differences are statistically significant.

The massive release of ROS in the early phase of reperfusion is considered one of the major factors contributing to I/R injury. In the present experiments, N-acetylcysteine (NAC), a potent ROS inhibitor, was used to investigate the role of oxidative stress in H/R-induced apoptosis and ERS. Total ROS detected with the fluorescent dye DCFH-DA indicated that H/R led to ROS accumulation in HK-2 cells (Fig. 3a). Meanwhile, the expression of apoptosis and ERS markers such as Bax, GRP78 and CHOP were significantly upregulated after H/R, while the expression of Bcl-2, a gene that inhibits apoptosis, was significantly decreased after H/R compared with the control group. And treatment with NAC reduced H/R-induced expression of proapoptotic and ERS proteins, with statistically significant differences (Bcl-2 0.8533 ± 0.008819 to 0.6133 ± 0.01453, Bax 1.470 ± 0.02887 to 2.397 ± 0.03712, GRP78 1.690 ± 0.02309 to 2.533 ± 0.04978, CHOP 1.947 ± 0.04256 to 2.777 ± 0.1453, n = 3, P < 0.05) (Fig. 3 b–e). These results demonstrated that H/R induced apoptosis and ERS in kidney cells through oxidative stress.

### C3G attenuated H/R-induced oxidative stress, apoptosis and ERS in vitro

**Figure 4:**
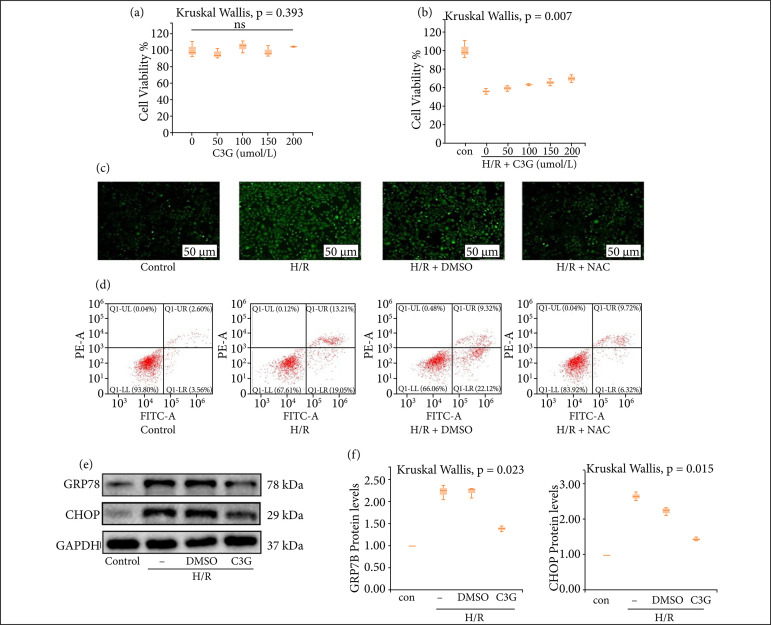
C3G attenuated H/R-induced oxidative stress, apoptosis and ERS in vitro. **(a)** HK-2 cells were treated with different concentrations (50, 100, 150, 200 µmol/L) of C3G for 24h, and then their cell viability was detected by CCK-8; **(b)** HK-2 cells were treated with different concentrations (50, 100, 150, 200 µmol/L) of C3G for 24 h to establish the H/R model, and then their cell viability was detected by CCK-8; **(c)** ROS production observed by fluorescence microscopy (×400); **(d)** Apoptosis was assessed by flow cytometry; **(e)** Protein blot analysis of GRP78, CHOP expression; **(f)** GRP78, CHOP protein expression was quantified by optical densitometry normalized to the expression of GAPDH from three independent experiments. Values are expressed as medians (IQR) on the box plot. P < 0.05 indicates that the differences are statistically significant. ns, no significance.

The CCK-8 assay was then used to determine the drug concentrations for in vitro experiments as shown in Fig. 4a, different concentrations of C3G were not significantly toxic to HK-2 cells under normoxic conditions. Next treated HK-2 cells with different concentrations of C3G for 24 h^19^ before establishing the cellular H/R model. The results showed that the optimal protection of cells against H/R damage was achieved by 200 µmol/L C3G (Fig. 4b). Therefore, 200 µmol/L was chosen as the standard for the subsequent in vitro experiments. At a concentration of 200 µmol/L, C3G pretreatment significantly reduced H/R-induced ROS production (Fig. 4c), and further reduced the number of apoptotic cells after H/R (Fig. 4d), and reversed the H/R-induced increase in ERS-related proteins, with statistically significant differences (GRP78 1.383 ± 0.04055 to 2.220 ± 0.09644, CHOP 1.439 ± 0.02906 to 2.623 ± 0.06642, n = 3, P < 0.05) (Fig. 4e, f). These indicated that C3G also has an effect on reducing I/R injury in HK-2 cells in vitro.

### C3G attenuated renal injury by inhibiting the JAK/STAT pathway

**Figure 5:**
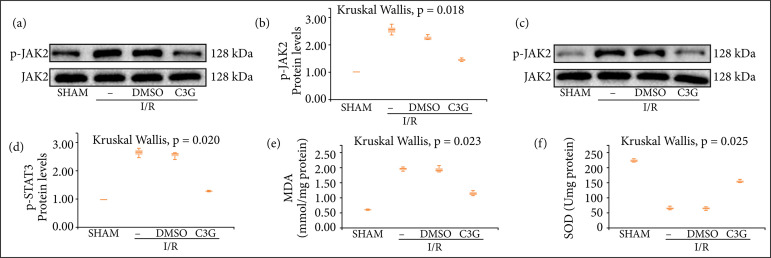
C3G attenuated renal injury by inhibiting the JAK/STAT pathway. (**a, c**) Protein blot analysis of protein expression of JAK2, p-JAK2, STAT3, p-STAT3; and (**b, d**) quantitative analysis of p-JAK2 and p-STAT3; (**e, f**) MDA, SOD production in mice. Values are expressed as medians (IQR) on the box plot. P < 0.05 indicates that the differences are statistically significant.

To further explore the potential mechanisms underlying the nephroprotective effects of C3G, the pathways that may be involved were investigated. C3G has been reported to attenuate breast cancer angiogenesis through inhibition of the STAT3/VEGF pathway(20). As shown in Fig. 5a-d, the levels of p-JAK2 and p-STAT3 were upregulated after I/R treatment compared with the I/R group, and C3G treatment could effectively inhibit the increase of their expression, and the difference was statistically significant (p-JAK2 1.443 ± 0.04333 to 2.537 ± 0.1155, p-STAT3 1.317 ± 0.01764 to 2.727 ± 0.1053, n = 3, P < 0.05). Moreover, compared with the I/R group, C3G significantly increased SOD activity and inhibited MDA production in I/R-injured mice, with statistically significant differences (MDA 1.133 ± 0.05239 to 1.947 ± 0.04096, SOD 153.7 ± 3.712 to 64.00 ± 3.606, n = 5, P < 0.05) (Fig. 5 en f). This suggested that C3G may attenuate I/R-induced oxidative stress via the JAK/STAT pathway, thereby attenuating apoptosis and ERS and achieving in vivo renal protection.

**Figure 6:**
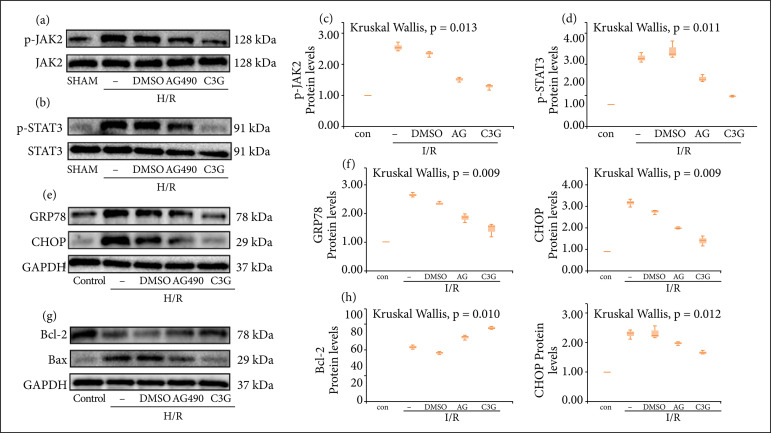
C3G attenuated renal injury by inhibiting the JAK/STAT pathway. (a, b) Protein expression of JAK2, p-JAK2, STAT3, p-STAT3 in cells of different treatment groups; (c, d) Quantitative analysis of p-JAK2 and p-STAT3; **(e)** Protein blot analysis of GRP78 and CHOP protein expression in the indicated groups; **(f)** Quantitative analysis of GRP78 and CHOP; **(g)** Representative protein blot analysis of Bax, Bcl-2 in the indicated groups. Quantitative analysis of Bax and Bcl-2.Values are expressed as medians (IQR) on the box plot. P < 0.05 indicates that the differences are statistically significant.

To test the above conjecture, further in vitro experiments were performed with a specific JAK inhibitor, AG490. It could be seen that both AG490 and C3G inhibited I/R-induced phosphorylation of JAK2 and STAT3 compared to the Control group, with statistically significant differences (p-JAK2 1.513 ± 0.04978 to 2.557 ± 0.08192, p-STAT3 2.180 ± 0.09849 to 3.103 ± 0.1224, n = 3, P < 0.05) (Fig. 6a-d). In addition, AG490 also inhibited the expression of downstream apoptosis and ERS-related proteins like C3G (Fig. 6e-h). C3G showed better anti-I/R injury activity, which may also be related to some other pathways. Overall, these results supported the above speculation that C3G attenuated renal injury by inhibiting the JAK/STAT pathway.

## Discussion

The aim of this study was to investigate whether C3G affects renal I/R injury and the possible mechanisms that mediate this effect. Firstly, a classical mouse I/R model was successfully established to verify the effect of C3G on I/R injury. The results showed that pretreatment with C3G attenuated I/R injury-induced renal tissue damage and dysfunction. Furthermore, C3G ameliorated I/R-induced apoptosis and ERS and inhibited phosphorylation of JAK2 and STAT3. What What was observed in vivo needs to be further validated in vitro. It was first demonstrated that the loss of cell viability induced by H/R in HK-2 cells could be reversed by C3G. Furthermore, elevated expression of the JAK/STAT pathway and increased oxidative stress-induced apoptosis and ERS were observed during H/R; however, C3G treatment reversed these changes induced by H/R. Moreover, the protective effect of C3G was similar to that of the JAK inhibitor AG490. Therefore, the findings suggest that C3G may be a potential drug for the treatment of renal I/R injury.

Recently, some studies have reported significant efficacy of C3G on I/R injury in the heart and liver^21^^-^ 23. Other study has provided new understanding of C3G metabolism in humans by 13C-labeled anthocyanin and isotope ratio mass spectrometry, which will inform the design of future clinical studies exploring the biological activity of these potentially important dietary compounds^24^. A growing evidence are suggesting that C3G may be a potential therapeutic agent for a variety of diseases including renal diseases^25^^,^ 26. Renal I/R injury is a major clinical challenge for clinicians in the perioperative period of renal transplantation as well as partial nephrectomy and is a major cause of AKI, resulting in structural and functional damage to the renal tubules. C3G has been reported to improve diabetic nephropathy by modulating glutathione pools, suggesting that C3G may have potential in the treatment of diseases of the kidney^26^^-^ 28. Another recent study reported that C3G attenuates breast cancer angiogenesis by inhibiting the STAT3/VEGF pathway^20^. In this study, it was demonstrated that C3G reversed the renal pathological damage caused by acute I/R injury and protect renal function. And consistent with the in vivo findings, the results of in vitro studies suggest that C3G protects HK-2 cells from H/R-induced damage.

Mechanisms of AKI induced by renal I/R include apoptosis^29^, ERS^30^, and oxidative stress^31^. Apoptosis is the genetically controlled, autonomous and orderly death of cells to maintain the stability of the internal environment. The endoplasmic reticulum is an organelle involved in protein modification and peptide chain folding. ERS is a stress mechanism initiated by external and internal factors that lead to excessive accumulation of misfolded or unfolded proteins and dysregulation of sterol and lipid levels^32^. Prolonged high levels of ERS can in turn induce apoptosis^33^. Both endoplasmic reticulum and apoptosis are important for cell function and survival. A large body of evidence suggests that ERS and apoptosis are essential steps in the pathogenesis of several renal diseases, including renal I/R injury^34^^,^ 35. Oxidative stress is a state in which the excessive production of free radicals, such as ROS, leads to an imbalance of oxidative and antioxidant effects in the body. The production of ROS during oxidative stress may modulate renal I/R injury by affecting ERS and thus apoptosis^36^. In the present study, it was found that H/R-induced apoptosis and ERS may be dependent on oxidative stress. The results showed that the expression of proapoptotic and ERS proteins such as Bax, GRP78, and CHOP were significantly upregulated after H/R, while the expression of apoptosis-inhibiting proteins such as Bcl-2 was decreased. Treatment with NAC to scavenge ROS reversed the H/R-induced increase in the expression of proapoptotic and ERS proteins. It was further demonstrated that C3G suppressed the increase in ROS levels after H/R. Therefore, the renoprotective effect of C3G may be achieved by inhibiting ROS generation induced by H/R injury.

The JAK2/STAT3 signaling pathway has been shown to play a critical role in injury and cancer in multiple systems^36^^-^ 38. Although the JAK2/STAT3 signaling pathway is mostly inactivated under basal conditions, it can be phosphorylated in response to extracellular stimuli, thereby affecting the transcription and expression of multiple genes involved in biological processes that further regulate cell growth, metabolism, differentiation, apoptosis, and oxidative stress^39^. A study surface that ischemic stimulation in the kidney rapidly activates the JAK2/STAT3 signaling pathway and that inhibition of its expression attenuates renal ischemic injury, associated with its reduction of apoptosis and mitochondrial damage^40^. However, there has been no surface role for the JAK2/STAT3 signaling pathway in the renal protective effects of C3G. The experiments were designed to determine whether C3G could prevent renal I/R injury through the JAK2/STAT3 signaling pathway. These results suggest that the renoprotective effect of C3G treatment is achieved by inhibiting the JAK2/STAT3 signaling pathway. AG490 is a JAK inhibitor that has been used to attenuate the JAK2/STAT3 signaling pathway in various studies(41-43). AG490 was used to explore the role of the JAK2/STAT3 signaling pathway in the renal protection of C3G. The results showed that the renoprotective effects of AG490 and C3G were the same, both through inhibition of the JAK2/STAT3 signaling pathway. both AG490 and C3G inhibited p-JAK2 and p-STAT3 expression and attenuated the ischemia-induced increase in Bax expression and decrease in Bcl-2 expression. In addition, both AG490 and C3G reversed the increase in ROS, SOD, and MDA due to I/R, suggesting that the JAK2/STAT3 signaling pathway may be involved in anti-apoptotic pathways and antioxidant effects.

## Conclusion

This study identified a protective role for C3G in renal I/R injury. It also found that C3G blocked the production of ROS through the JAK/STAT pathway, thereby blocking renal apoptosis and ERS protein expression. Overall, these results suggest that C3G is a potential therapeutic agent for the treatment of renal I/R injury.

## Data Availability

The data will be available upon request.
